# Programmable Topological Light via Coherent Beam Combination

**DOI:** 10.1021/acsphotonics.6c01419

**Published:** 2026-09-04

**Authors:** Fedor Chernikov, William Kerridge-Johns, Konstantin K. Bobkov, Johan Nilsson, Michalis N. Zervas, Ben Mills

**Affiliations:** Optoelectronics Research Centre, 7423 University of Southampton, Southampton SO17 1BJ, U.K.

**Keywords:** Structured light, topological photonics, coherent beam combination, optical skyrmions

## Abstract

Topologically structured optical fields exhibit rich physical properties and offer new opportunities for information encoding and light-matter interaction. In this work, we propose a general and scalable framework for generating and reconfiguring topological light using coherent beam combination (CBC). By treating the CBC system as an electronically addressable optical phased array, we demonstrate that the topology of the optical field becomes a programmable parameter. As a representative example, we focus on optical skyrmions and present an experimental demonstration of deterministic and dynamic switching between Bloch-type, Néel-type, and antiskyrmion textures using a 7-channel Yb-doped fiber master oscillator power amplifier CBC system. This approach establishes a scalable and programmable route toward high-power topological vector fields and reconfigurable structured light.

## Graphical abstract

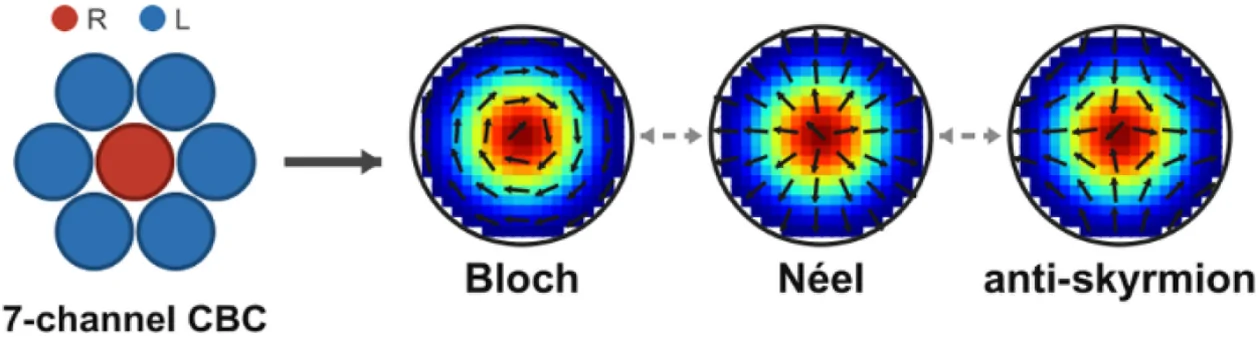

## Introduction

Topologically structured optical fields, with spatially varying polarizations, have attracted significant interest for both fundamental physics and a wide range of potential applications.^1–4^ Among these, optical skyrmions have emerged as a distinct class of topologically protected quasiparticles^5^ whose vector-field configurations emulate those found in particle physics.^6^ The topological state of an optical skyrmion is quantified by a skyrmion number, an integer invariant that is preserved under continuous perturbations.^7^ This combination of spatially structured polarization and topological protection has enabled emerging applications in optical information encoding,^8,9^ advanced material processing,^10,11^ among others.^12–14^

Optical skyrmions were first generated in the evanescent field of surface waves,^15^ and later in tightly focused free-space fields.^16^ They were subsequently constructed in the paraxial regime using Stokes-vector-defined vector beams.^17^ However, these early demonstrations primarily produced fixed skyrmionic textures and did not enable tunable topological configurations. More recently, tunable optical skyrmions have been demonstrated in free space using holographic beam-shaping systems,^18,19^ mechanically tunable polarization optics^20^ and a metafibre platform.^21^ Together, these approaches have enabled the generation of diverse topological textures, including Néel-type and Bloch-type skyrmions, antiskyrmions, and bimerons.^22^ They have also established important capabilities in high-fidelity field generation, propagation stability and controlled transformation between topological states. Recent studies have further explored optical skyrmions for free-space information transmission, demonstrating their topological resilience over a range of atmospheric turbulence strengths.^23–25^ In parallel, spatially structured polarization fields have shown potential for controlling light-matter interactions in laser material processing.^11,26^ These developments motivate interest in sources capable of generating structured polarization fields at elevated optical powers while enabling rapid reconfiguration of their topology. Existing approaches, however, do not generally target power scalability or fast, programmable, electronically controlled switching among multiple topological textures, especially not in combination.

Coherent beam combination (CBC) through active phasing of tiled optical beamlets provides a complementary route toward achieving this combination of capabilities. In such a CBC system, multiple independent beams (beamlets) are spatially overlapped and phase-locked to generate a single output,^27–29^ allowing the transverse field to be dynamically shaped by actively controlling the relative phases, amplitudes or polarizations of the constituent beamlets.^30–32^ Although CBC is conventionally used to scale optical power, its inherent modularity and compatibility with fast electronic control make it naturally suited for synthesizing complex spatial fields. In free-space optical propagation and communication, the adjustable wavefront provided by the CBC has been exploited to compensate for turbulence-induced aberrations.^33–35^ CBC has also been used to generate structured light, but this has been largely confined to creating cylindrical vector beams with simple radial or azimuthal polarization topologies.^32,36,37^ To date, its potential for generating more sophisticated, dynamically reconfigurable topological textures, such as optical skyrmions, remains unexplored.

In this work, we propose and demonstrate CBC as a unified framework for generating optical skyrmionic textures. First, we establish the general principles underlying the formation of skyrmionic textures. Each constituent beamlet is treated as a programmable unit which can, in principle, provide per-channel electronic access to the constituent field components, enabling independent and continuous control of phase, amplitude and polarization. In the present work, we primarily exploit phase control, while the amplitude and polarization states are preset. Based on this framework, we describe the formation principle of fundamental skyrmionic textures such as Néel-, Bloch-, and antiskyrmions, as well as higher-order textures that emerge from more complex combinations of spatial phase and polarization. We then present an experimental realization using a 7-channel CBC system, in which we generate Néel-type, Bloch-type, and antiskyrmion textures in the central lobe of the combined beam and demonstrate dynamic switching between these topological configurations through electronically controlled phase adjustments. These results extend the functionality of CBC beyond conventional power scaling and wavefront control by introducing programmable polarization topology as an additional degree of freedom. The approach establishes a pathway toward power-scalable and dynamically reconfigurable structured-light sources, whose tunable topologies hold high potential for applications in robust free-space optical communication, advanced laser material processing and the investigation of high-power topological optical fields.

## Results and Discussion

### Stokes Vector Optical Skyrmions

An optical skyrmion is formed through a continuous topological mapping from a three-component vector field defined in a parametric space onto a two-dimensional real spatial domain.^38^ In optics, this parametric space is provided by the three-dimensional Stokes-vector space

1 $$S\left(x,y\right)=\left(S_{1}\left(x,y\right),\;S_{2}\left(x,y\right),\;S_{3}\left(x,y\right)\right)$$

which describes the local polarization state of light on the Poincaré sphere, while the real space corresponds to the transverse (*x*,*y*) plane of the optical field. The topology of this mapping is quantified by the skyrmion number:

2 $$s=\frac{1}{4\pi }\iint_{\sigma }n\cdot \left(\frac{\partial n}{\partial x}\times \frac{\partial n}{\partial y}\right)dxdy$$

where $n\left(x,y\right)=\frac{S\left(x,y\right)}{\left|S\left(x,y\right)\right|}$ is the local unit Stokes vector, and *σ* denotes the spatial region over which the skyrmionic texture is defined. The integer value of *s* represents the number of times the polarization vector wraps around the Poincaré sphere.

In practice, an optical skyrmion can be realized as a superposition of two orthogonally polarized spin components, each associated with a distinct spatial mode:

3 $$\left|\psi \right\rangle =u_{0}\left|0\right\rangle +e^{i\theta }u_{1}\left|1\right\rangle$$

where |0⟩ and |1⟩ denote two orthogonal optical polarizations, *u*_0_ and *u*_1_ are orthogonal spatial modes, and *θ* is the relative phase between the two components. Here we take |0⟩ and |1⟩ to be |*R*⟩ and |*L*⟩ states (i.e., right- and left-circularly polarized), and *u*_0_ and *u*_1_ to be Laguerre-Gaussian (LG) modes, each carrying an azimuthally varying phase term $e^{il\phi }$ , where *ϕ* is the azimuthal angle in cylindrical coordinates, corresponding to orbital angular momentum (OAM) of charge l . When the OAM indices of the two modes differ, i.e., $l_{0}\neq l_{1}$ , their superposition leads to a spatially varying polarization distribution across the transverse plane, resulting in a nontrivial Stokes-vector texture. In particular, the superposition of an LG mode with l=0 in the |*R*⟩ state and an LG mode with |l|=1 in the |*L*⟩ state yields a skyrmionic texture: for l=−1 the resulting texture corresponds to a Néel- or Bloch-type skyrmion (depending on the phase *θ*), whereas l=+1 yields an antiskyrmion.

Therefore, our approach for generating skyrmionic textures using CBC is to construct two orthogonal spatial components approximating LG modes with l=0 and l=±1 with orthogonal circular polarizations. Recently, it has been demonstrated that tiled-aperture CBC systems can approximate a wide class of structured beams, including LG modes carrying OAM, through phase-only control of the relative phases between individual channels.^39^ Building on this capability, we employ the CBC system as a discrete programmable optical phased array to synthesize the orthogonal spatial mode components required for optical skyrmion formation.

### Optical Skyrmions through Tiled Aperture CBC

We begin our theoretical model by considering the inner 19 beamlets (each described by a Gaussian beam profile) of a 37-channel CBC system arranged in a hexagonal close-packed (HCP) geometry, as shown in Figure 1a. When these 19 beamlets are in-phase (Figure 1b) and prepared in the |*R*⟩ state, the resulting far-field distribution takes the form shown in Figure 1c, with the central region closely approximating the l=0 LG mode and retaining the local spin eigenstate defined by the |*R*⟩ input channels. To approximate the l=±1 LG mode, the outer ring of 18 beamlets, shown in the Figure 1d, is prepared in the orthogonal |*L*⟩ state and assigned a linearly increasing azimuthal phase, as demonstrated in the Figure 1e. Starting from a reference beamlet with phase 0*π*, the phase increments by *π*/9 between neighboring beamlets, completing a full 2*π* wrap around the ring. This phased ring produces a far-field pattern whose central region closely resembles the l=±1 LG mode as shown in Figure 1f, where a clockwise phase increase corresponds to l=+1 and a counterclockwise increase corresponds to l=−1 , while maintaining the |*L*⟩ state defined by the outer beamlets.

**Figure 1:**
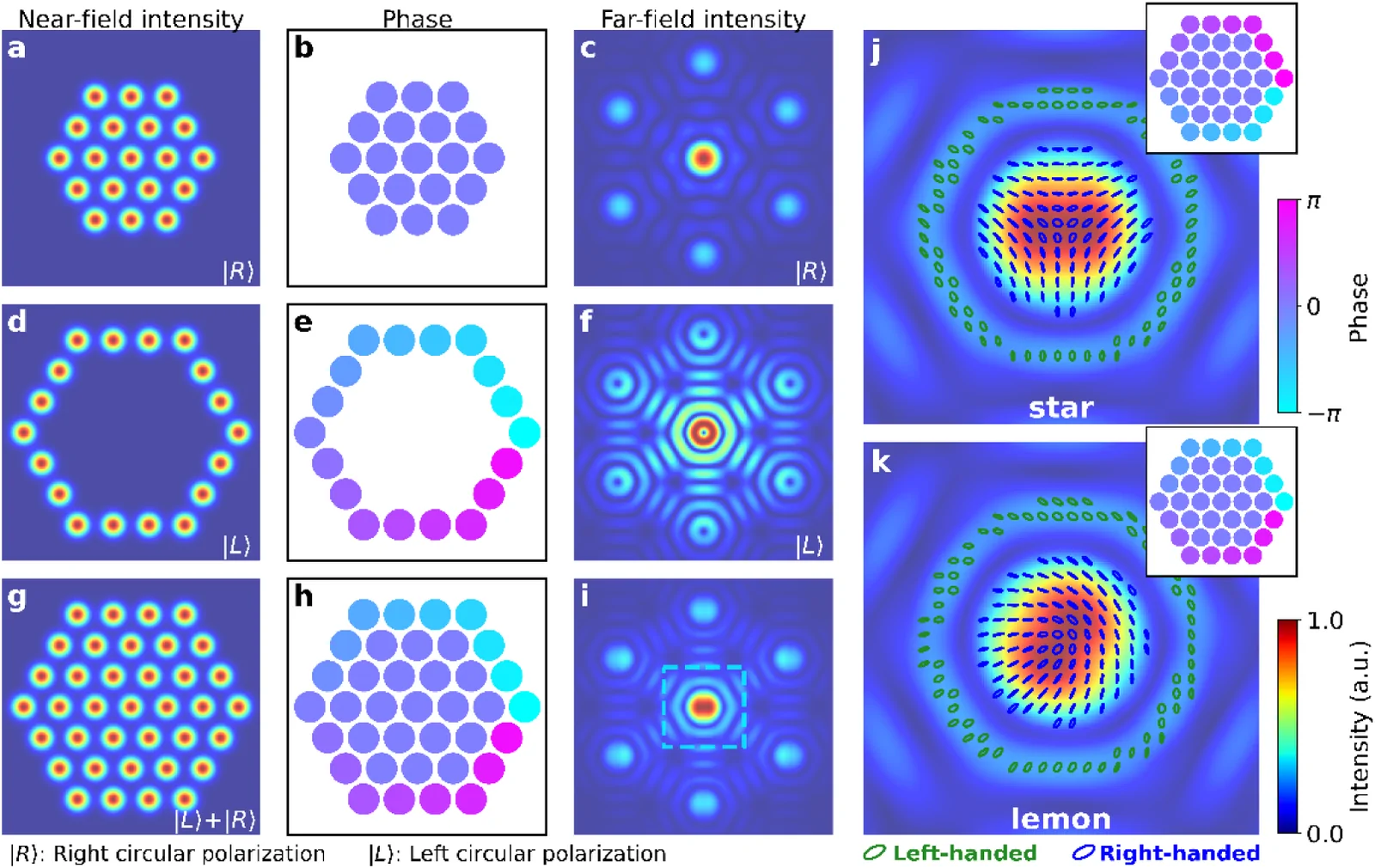
Schematic of skyrmionic texture formation using CBC. **a**–**c**, Near-field intensity, near-field phase, and far-field intensity distributions of the inner 19 fibers in a 37-channel CBC array. **d**–**f**, Near-field intensity, near-field phase, and far-field intensity distributions of the outer 18 fibers. **g**–**i**, Near-field intensity, near-field phase, and far-field intensity distributions of all 37 fibers. **j**, **k**, Enlarged views of the far-field intensity distribution over the spatial region highlighted, with the region corresponding to (**k**) highlighted in (**i**), and polarization ellipses overlaid. The polarization ellipses are defined by the direction of the phase wrapping of the outer 18 fibers, as illustrated in the corresponding insets, forming star-type (**j**) and lemon-type (**k**) structures, respectively.

With both l=0 and l=±1 components constructed, we next consider their coherent superposition. The resulting combined near-field intensity and phase distribution are shown in Figure 1g and h, respectively. The phase distribution of the superimposed field contains both the in-phase central contribution from the inner beamlets and the azimuthal phase ramp associated with the outer ring. The overall far-field intensity pattern is shown in Figure 1i and exhibits a spatially varying polarization structure across the transverse plane. Practical implementations of CBC necessitate space between beamlets and incomplete filling of optical intensity across the tiled aperture. We include this feature in our theoretical consideration, leading to the typical spreading of optical power into satellite lobes. The dominant power fraction is contained in the main central lobe, which is where we focus our polarization analysis, and improved practical designs can enhance this fraction at the expense of reduced background.

Figure 1j–k shows a magnified view of the same spatial region, with the region corresponding to Figure 1k highlighted in Figure 1i, with the local polarization state overlaid on the corresponding intensity distribution and an inset illustrating the relative phase between the fibers. The phase array, shown in the inset of Figure 1j, corresponds to the superposition of l=0 and l=+1 LG-like beams, producing the characteristic *star*-type polarization distribution in the central region. Conversely, Figure 1k presents the case where the l=0 and l=−1 LG-like beams are superposed, resulting in a *lemon*-type polarization distribution in the central spot.

To quantitatively confirm the skyrmionic nature of the generated field and to demonstrate the tunability of the skyrmionic topological states through phase-only control, we compute the corresponding skyrmion number and examine the associated Stokes-vector texture. Figure 2 presents a combined intensity-polarization-topology representation of the generated vector fields. The top row shows the central region of the combined far-field intensity pattern, with the local polarization state overlaid. The bottom row presents the corresponding Stokes-vector maps, which directly visualize the mapping of the transverse plane onto the Poincaré sphere. The insets between the two rows display the relative phase distributions that give rise to the particular far-field patterns under consideration.

**Figure 2:**
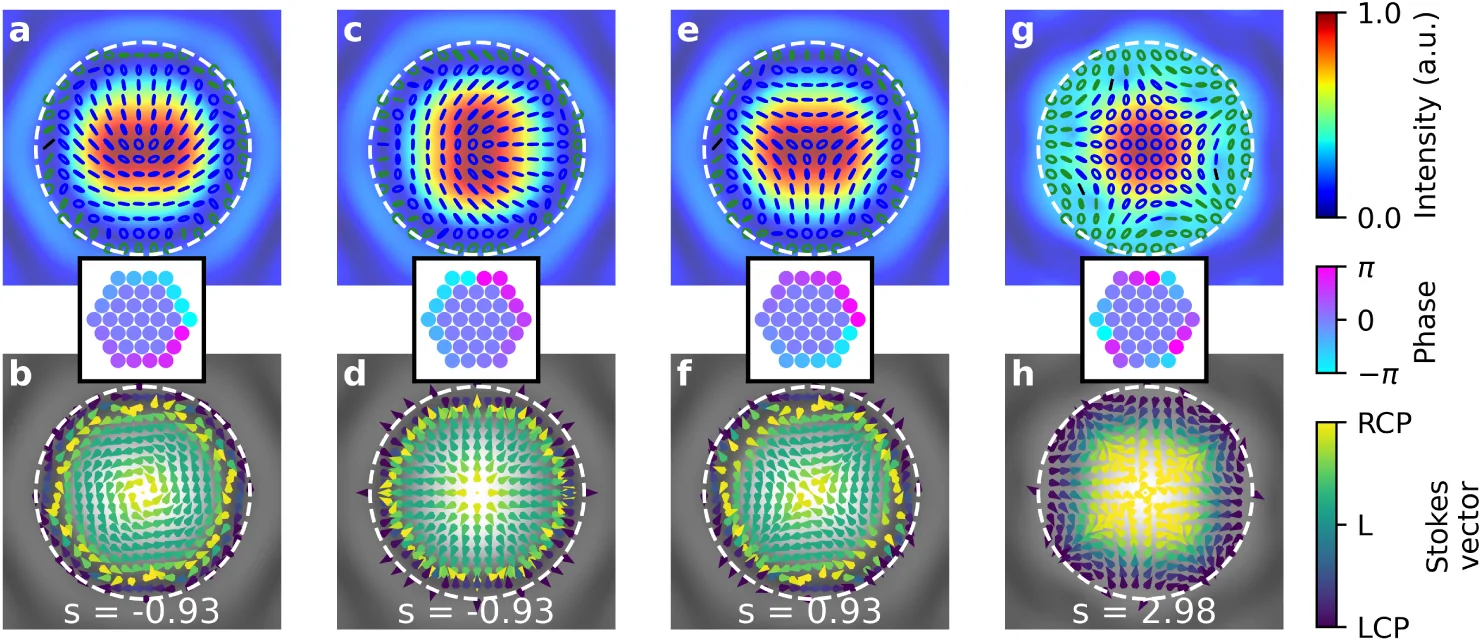
Tunability of optical skyrmion using programmable phase adjustments in CBC. The top row shows the central region of far-field intensity distribution with polarization ellipses overlaid (where blue and green denote right- and left-handed polarization), and the bottom row presents the Stokes vector maps. The insets in the middle row display the associated phase distributions used to generate the different skyrmionic textures. **a**, **b** Bloch-type skyrmion. **c**, **d** Néel-type skyrmion. **e**, **f** antiskyrmion. **g**, **h,** Higher order antiskyrmion texture. The skyrmion number s is indicated at the bottom of each Stokes vector map. White circles indicate the common circular integration region used for calculation of the skyrmion number.

Figure 2a demonstrates a *lemon*-type polarization distribution. The corresponding Stokes-vector map shown in Figure 2b exhibits a clear vortex spiral texture, characteristic of a Bloch-type skyrmionic texture. The calculated skyrmion number is *s* = −0.93 (with the integral taken over the circular aperture indicated by the white dashed line), which is close to the expected theoretical value of −1 for this class of skyrmion.

A major attraction of the CBC system is that it enables rapid phase control of every constituent beamlet. To transform the Bloch-type texture into a Néel-type, a relative phase delay of *π*/2 must be introduced between the two constituent orthogonal spatial mode components forming the skyrmion. In the CBC system, this is achieved by applying an additional *π*/2 phase shift to the outer ring of beamlets. Figure 2c shows that, as a result, the local *lemon*-type polarization orientation rotates by *π*/2 radians. This operation preserves the original *π*/9 phase difference between adjacent beamlets while effectively modifying the relative phase between the inner and outer subarrays. The corresponding Stokes-vector map, shown in Figure 2d, exhibits a hedgehog structure, characteristic of a Néel-type skyrmion, with the skyrmion number remaining unchanged, *s* = −0.93, confirming that the phase-only transformation alters the skyrmion helicity without modifying its topological charge.

To complete the set of fundamental skyrmionic textures, the direction of the azimuthal phase ramp is reversed from counterclockwise (inset of Figure 2a) to clockwise (inset of Figure 2e), causing the polarization distribution to transform from *lemon*-type to *star*-type as shown in Figure 2e. The corresponding Stokes-vector map, shown in Figure 2f, now exhibits a saddle-like structure. In this case, the skyrmion number changes sign while preserving its magnitude, confirming the formation of an antiskyrmion with *s* = 0.93.

It should be noted that the polarization texture, and correspondingly the Stokes-vector field, is not perfectly continuous when outer ring generates LG_01_-like mode. Along the radial direction, the polarization evolves from right-circular at the center, through linear, and then through a region of elliptical states before reaching left-circular polarization at the outer edge. Although this may appear to leave part of the Poincaré sphere uncovered, the outer regions contain both elliptical and left-circular states, whose combined contribution brings the integrated skyrmion number close to unity. The sensitivity of the skyrmion number to the size of the integration aperture is examined in Supporting Information, Section 1. We attribute the imperfect continuity to the incomplete spatial overlap between the constituent LG_00_- and LG_01_-like modes, which could be improved by, for example, amplitude apodization across the array, although this lies beyond the scope of the present work.

To further demonstrate the phase-only shaping capability of the CBC system, we take the antiskyrmion configuration and its associated phase distribution as a starting point. Instead of applying a *π*/9 phase increment with an overall 2*π* azimuthal wrap, we now impose a total phase wrap of 6*π*, corresponding to three full 2*π* phase wraps around the ring. As a result, the phase increment between adjacent beamlets becomes *π*/3. Following the same reasoning as above, this configuration effectively realizes the superposition of l=+3 and l=0 components. The corresponding local polarization distribution and Stokes-vector map are shown in Figure 2g and h. The characteristic star-type polarization structure and the associated saddle-like Stokes-vector texture are preserved, but now appear in a higher-order form with a skyrmion number of *s* ≈ 3, calculated over the same spatial region as for the lower-order skyrmions. The polarization evolution is also visibly closer to continuous in this case: both the polarization ellipses and the Stokes-vector map show a smoother progression from right- to left-circular polarization, reflecting the improved spatial overlap between the constituent modes at higher OAM charge. This simple phase adjustment demonstrates a highly flexible route toward tunable and higher-order skyrmion formation using CBC. A similar approach also gives access to bimeronic textures, which are topologically equivalent to skyrmions and differ only by a rotation of the Poincaré sphere before projection onto the 2D plane. Reassigning the two constituent components to form orthogonal linear states moves the poles of the texture onto the equator and produces bimerons of identical skyrmion number, as detailed in Supporting Information, Section 2. The achievable order of the skyrmionic and bimeronic textures is, however, bounded by the emitter plane implementation. Because the ${\mathrm{LG}}_{0l}$ -like mode is generated from a discrete number of beamlets, the azimuthal sampling coarsens as l increases, until the resulting wavefront no longer forms a faithful approximation to the continuous helical front of the ${\mathrm{LG}}_{0l}$ mode. This sets an upper limit on the attainable charge, which can in principle be raised by increasing the number of emitters, although spatial matching between the LG_00_- and ${\mathrm{LG}}_{0l}$ -like modes must then also be maintained (see Supporting Information, Section 3).

Since the skyrmionic texture arises from the interference of multiple beamlets, it is important to establish the stability of the resulting polarization distribution under free-space propagation. Figure 3 shows the simulated skyrmion number of a freely propagating array as a function of propagation distance *z* and of the radius of the circular aperture over which the skyrmion number is evaluated. The calculation is performed for three configurations in which the outer ring of fibers generates an ${\mathrm{LG}}_{0l}$ -like mode with l=1 , 2 and 3, as shown in Figure 3a–c, respectively. The three cases differ only in the phases applied to the outer fibers, the array itself remains unchanged. Because beams diffract and expand as they propagate, a fixed physical aperture radius *r* would enclose a different fraction of the beam at each *z*. We therefore express the aperture in the angular coordinate *θ* = *r*/*z* : this removes the trivial linear magnification of the diffracting beam, so that the far-field texture becomes stationary in *θ* and the skyrmion content can be compared on equal footing across all propagation distances.

**Figure 3:**
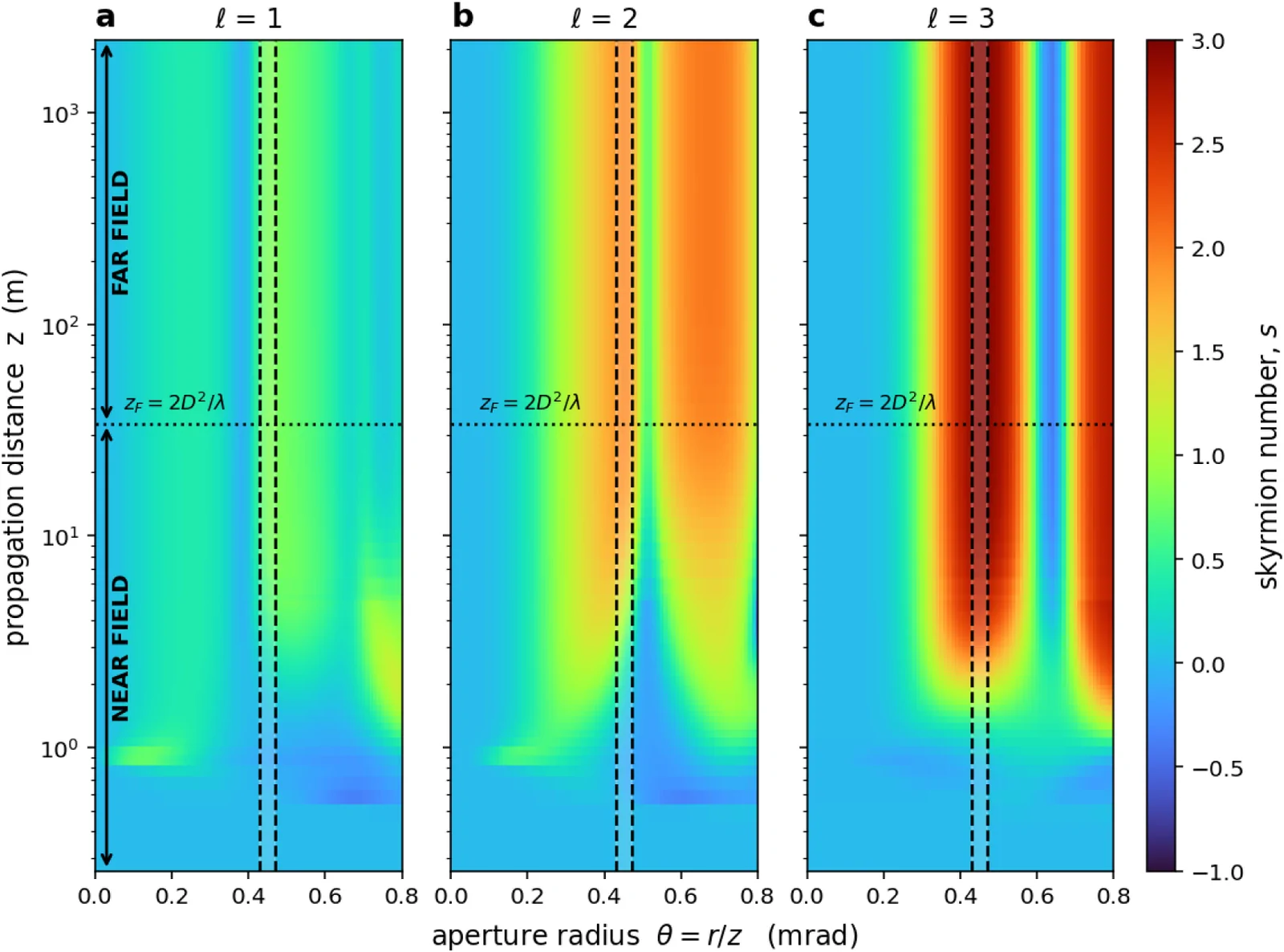
Skyrmion number *s* as a function of angular aperture radius *θ* = *r*/*z* and propagation distance *z* when the outer ring of a 37-fiber CBC array forms an ${\mathrm{LG}}_{0l}$ -like mode with vortex charge (a) l=1 , (b) l=2 , (c) l=3 . The near-field and far-field regions are separated by the Fraunhofer distance *z*_*F*_ = 2*D* ^2^/*λ*. The dashed lines mark the same range of angular aperture radii in all three panels, within which the skyrmion number reaches *s* ≈ 1, 2 and 3, as expected for the corresponding OAM charge, and retains that value throughout the far field.

In the near field the beamlets remain spatially separated and the field is locally either left- or right-circularly polarized, so no continuous texture exists and *s* ≈ 0. As the beamlets diffract and overlap, their interference populates the intermediate polarization states and a nontrivial polarization texture develops. Beyond the Fraunhofer distance *z*_*F*_ = 2*D* ^2^/*λ*, where *D* is the full diameter of the emitting array, the diffraction pattern becomes self-similar and the skyrmion number no longer changes with further propagation, confirming that the texture is topologically stable once formed. The dashed lines in Figure 3a–c mark a common range of angular aperture radii, identical in all three panels, within which the skyrmion number reaches *s* ≈ 1, 2 and 3 for l=1 , 2 and 3, respectively, and retains that value throughout the far field. A single fixed analysis region therefore returns the expected topological charge for all three configurations. This is a practical advantage for rapid switching: the topological state is reconfigured by phase alone, with no adjustment required to the aperture used to evaluate the skyrmion number, or to any other component of the CBC system.

To conclude, our tiled-aperture CBC configurations for generating high-quality optical skyrmionic textures are conceptually straightforward and, importantly, offer significant practical advantages for experimental implementation. In this work, we deliberately restrict the individual beamlets to pure |*L*⟩ or |*R*⟩ polarization states, which are readily achievable and robust experimentally. While this approach does not preclude the use of more general polarization states, such as elliptical polarizations, the practical demands on the CBC system would be substantially increased in such cases. The same applies to alternative assignments of the mode roles between the fiber groups: the complementary arrangement, in which the inner fibers form the ${\mathrm{LG}}_{0l}$ -like mode and the outer ring the LG_00_-like reference, is viable at low order but becomes increasingly difficult to sample as the charge is raised, as shown in Supporting Information, Section 4. A systematic investigation of these extended CBC configurations, as well as a comparative analysis of their performance, lies beyond the scope of the present work.

## Experimental Demonstration

An experimental demonstration was carried out using a 7-channel Yb-doped fiber (YDF) master oscillator power amplifier CBC system to validate the proposed concept for generating programmable optical skyrmions. Optical skyrmion formation followed the framework described in the previous section, in which two distinct groups of fibers contribute to LG_01_- and LG_00_-like modes with orthogonal polarization spin components. In the 7-channel CBC system, the LG_00_-like mode was provided exclusively by the central fiber which was also used as a reference to eliminate arbitrary phase offsets (i.e., the phases of the outer fibers are defined relative to the central one). The LG_01_-like mode was formed by the collective contribution of the six outer fibers, achieved by imposing a relative phase increment of *π*/3 between adjacent outer fibers, completing a full 2*π* phase wrap across the array. Controlled relative phase offsets between the central fiber and the outer-fiber group, configured with either clockwise or counterclockwise azimuthal phase ramp, gave rise to the different skyrmionic textures. The experimental implementation of this concept is schematically shown in Figure 4.

**Figure 4:**
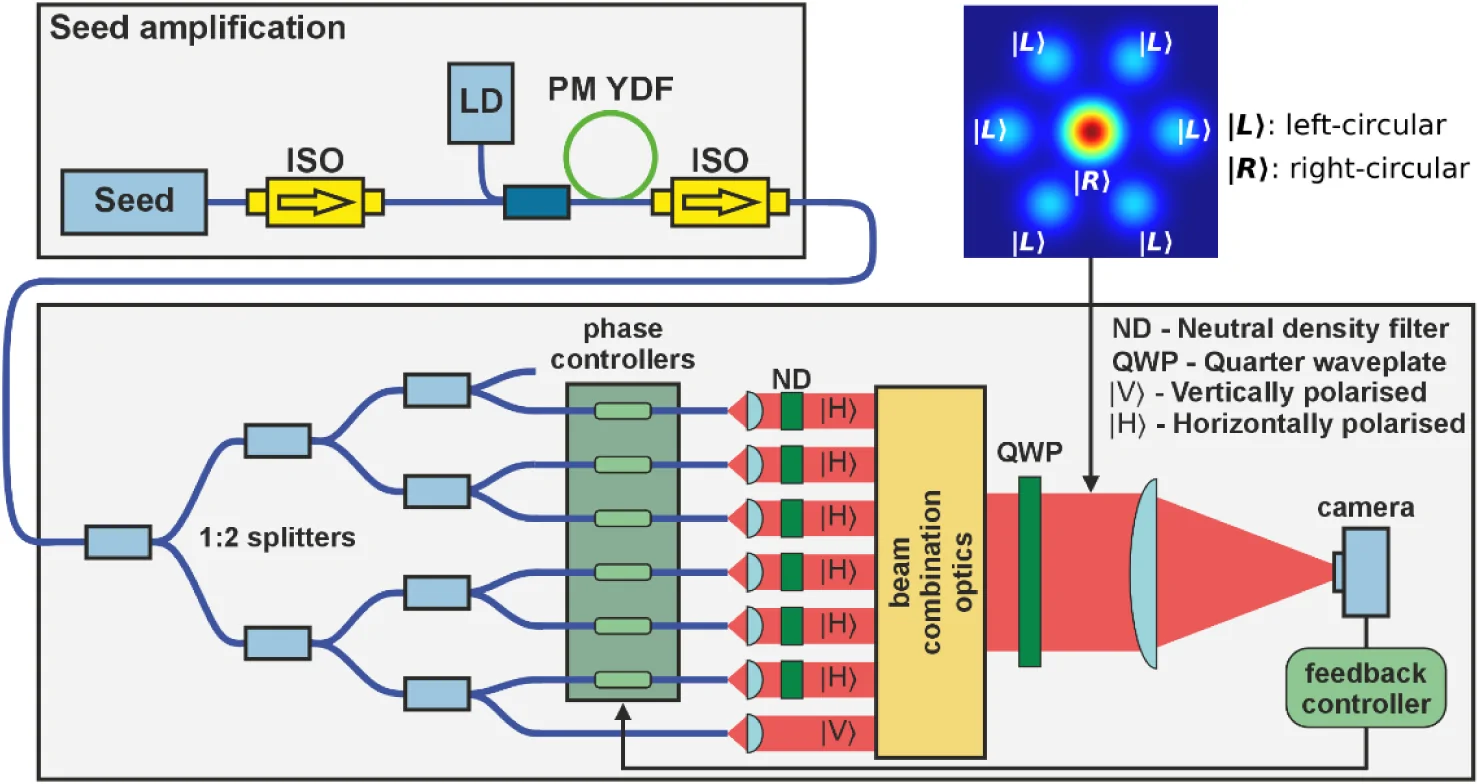
Schematic of the experimental 7-channel Yb-doped fiber amplifier CBC system to generate optical skyrmionic textures. LD: laser diode; PM YDF: polarization-maintaining Yb doped fiber; ISO: optical isolator.

A single-frequency fiber laser operating at 1070 nm (NKT Koheras MIKRO, line width < 0.1 kHz) was used as the seed source. The seed was amplified to an output power of approximately 500 mW using a laser-diode (LD) pumped fiber amplifier and subsequently split into eight channels, each with optical powers of approximately 40 mW. Seven of these channels were used to form a tiled-aperture HCP CBC array, while the remaining channel was left unused. All fibers and components were polarization-maintaining (PM), ensuring stable linear polarization throughout the fiber system. The measured polarization extinction ratio exceeded 15 dB, which is sufficient for efficient CBC operation.

The seven fiber outputs designated for combination were first collimated, after which their relative power levels and polarization states were adjusted prior to free-space combination in the far field, where the individual beams fully overlap. Within the tiled aperture, the six outer fibers were configured in a horizontal linear polarization state, denoted as |*H*⟩, while the central fiber was physically rotated to produce vertical linear polarization, referred to as |*V*⟩. Power apodization of the outer channels was achieved by inserting neutral density filters (ND, ND = 0.5), thereby balancing the relative contributions of the LG_01_-like mode formed by the outer six fibers and the LG_00_-like mode associated with the central fiber. This resulted in an approximate power ratio of 1/3 between each outer fiber and the central fiber. This power ratio balances the completeness of the polarization texture against the intensity distribution of the combined beam and its effect on the skyrmion number is examined numerically in Supporting Information, Section 5. The beams were subsequently spatially reconfigured into an HCP tiled array geometry and passed through a quarter-wave plate (QWP), converting the |*H*⟩ and |*V*⟩ polarization states into |*L*⟩ and |*R*⟩ circular polarization states, respectively. Once prepared, the polarization states and relative power levels remained stable throughout the experiment. The combined beam was then focused using a focusing lens and recorded on a CMOS camera for closed-loop control.

The closed-loop control system served two distinct purposes. First, it compensated for random phase differences between the channels originating from the separate propagation paths, which constantly drift from environmental changes, thereby maintaining a fixed relative phase difference across the CBC array. Second, it enabled the deliberate and programmable establishment of predefined relative phase differences between the channels, which were used to generate different optical skyrmionic textures. This control was implemented using a neural network (NN) based scheme employing the differential method described in our previous work.^40^ The NN inferred the voltage changes required to be applied to the phase controllers in order to maintain a specified relative phase difference. The output of the NN was consequently applied to in-house-built piezoelectric fiber stretchers, which served as phase actuators, ensuring that the desired phase corrections were accurately applied. The phase actuators were incorporated into the six outer fibers, while the central fiber was left free-running and served as a phase reference, with all outer-fiber phases defined relative to it.

As a result of the experiment, three distinct skyrmionic textures were observed: Bloch-type, Néel-type, and antiskyrmion. Figure 5 shows the central region of the combined beam, with the top row presenting experimental results recorded on the camera and the bottom row showing the corresponding theoretical predictions, with Stokes vector maps and polarization ellipses patterns overlaid.

**Figure 5:**
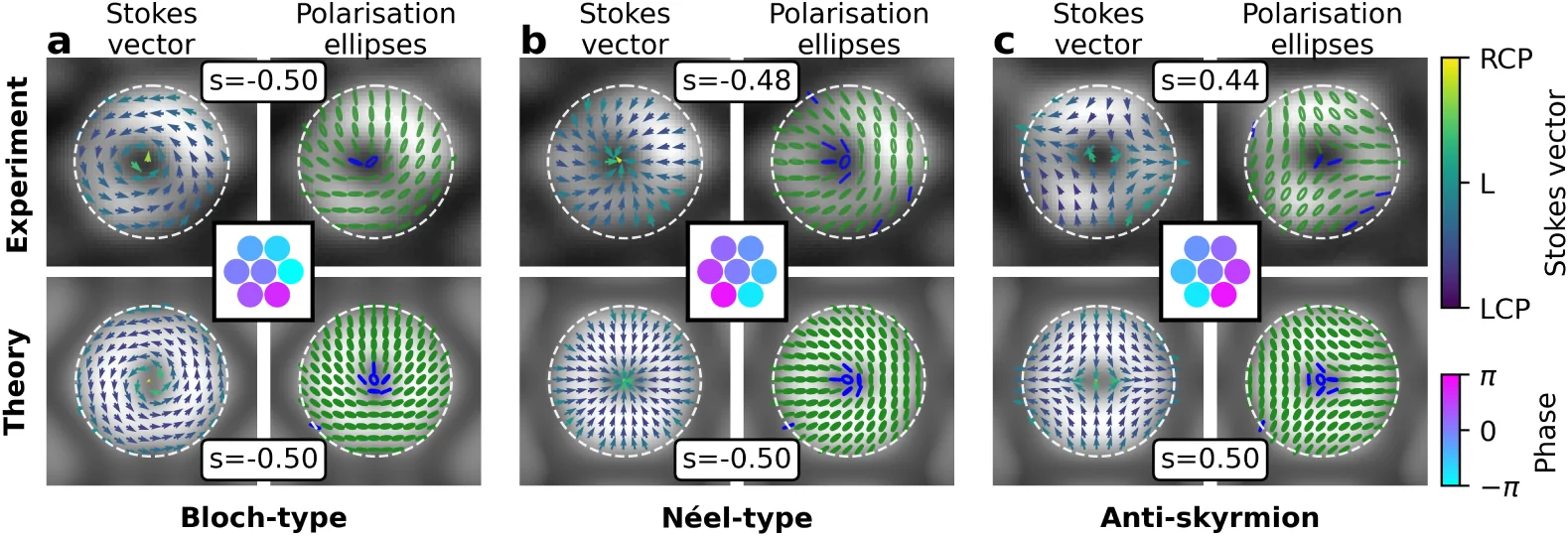
Experimental (top row) and theoretical (bottom row) Stokes vector maps and corresponding polarization ellipses (where blue and green denote right- and left-handed polarization) for (a) Bloch-type skyrmion, (b) Néel-type skyrmion, and (c) antiskyrmion. Insets illustrate the relative phase difference used to realize each skyrmionic texture. White circles indicate the common circular integration region used for calculating the skyrmion number.

In all three cases, the free-running phase of the central fiber was used as the zero-point phase reference. To realize the Bloch-type skyrmionic texture shown in Figure 5a, the phases of the six outer fibers were arranged such that the leftmost outer fiber was assigned a phase of 0*π* (i.e., in phase with the central fiber), and successive phase increments of *π*/3 were applied in the counterclockwise direction. This counterclockwise phase wrap, antiparallel to the polarization spin direction of outer fibers, effectively produced an LG_01_-like mode with l=−1 . As a result, a characteristic *lemon*-type polarization ellipse pattern was observed, while the Stokes vector map exhibited a vortex structure, confirming the formation of a Bloch-type skyrmionic texture. The experimental observation is in excellent agreement with the theoretical results shown in the bottom row.

To transform the Bloch-type skyrmionic texture into a Néel-type texture, an additional phase shift of *π*/2 was applied uniformly to the phases of the outer fibers. With the phase distribution shown in the inset Figure 5b, the polarization ellipses retained their *lemon*-type pattern, albeit rotated by *π*/2 radians, while the Stokes vector map evolved into a hedgehog-like structure characteristic of a Néel-type skyrmionic texture. The experimental results showed good agreement with theoretical predictions shown in the bottom row of Figure 5b and illustrate how phase adjustments enable controlled switching between distinct skyrmionic textures.

Finally, the antiskyrmion texture was obtained by reversing the direction of the 2*π* phase wrap from counterclockwise to clockwise. In this case, the phase wrapped in a direction parallel to the polarization spin of the outer fibers, making the collective contribution of the outer fibers equivalent to the LG_01_-like mode with l=+1 . This led to a transformation of the polarization ellipses from *lemon*-type to *star*-type pattern. Simultaneously, the corresponding Stokes vector map exhibited a saddle-like structure, confirming the formation of an antiskyrmion texture. This demonstrates a straightforward form of topological switching achieved solely through controlled phase adjustments.

The skyrmion numbers were calculated using the integral defined in eq 2, evaluated over the circular region shown in Figure 5. From the experimental data, the skyrmion numbers obtained for the Bloch-type, Néel-type, and antiskyrmion textures are −0.50, −0.48, and 0.44, respectively, compared with the corresponding theoretical values of −0.50, −0.50 and 0.50. While the experimentally obtained values are reduced relative to the ideal magnitude of unity, they remain close to the theoretical predictions, confirming that the essential skyrmionic topology is successfully realized.

The deviation from the ideal integer value of |1| indicates that the polarization states do not fully cover the Poincaré sphere. This behavior is primarily attributed to the geometry and discrete nature of the 7-channel CBC array, in which the LG_01_-like mode is synthesized from only six outer fibers, while the LG_00_-like mode is provided exclusively by the central fiber. Consequently, the effective spatial extents of the LG_01_-like and LG_00_-like modes are not perfectly matched and achieving an optimal spatial and power balance between the two contributions is inherently constrained in the 7-channel CBC array case.

These discretization-related limitations are naturally alleviated in CBC systems with a larger number of channels. For example, in a theoretically considered 37-channel configuration, the LG_01_-like and LG_00_-like modes can be formed from 18 outer and 19 inner fibers, respectively, leading to improved modal matching and a more natural power balance. Such configurations therefore provide a clear pathway toward higher-fidelity skyrmionic textures, while the present 7-channel system serves as a conceptually simple and experimentally accessible proof of principle.

Beyond the improvement in fidelity, a larger channel count also provides a direct route to power scaling, with each additional emitter contributing its own power to the combined output. Another route to power scaling is to raise the power carried by each channel. While the present work was performed at relatively low power, comparable differential phase-noise performance has been reported for this system at up to 200 W per channel.^40,41^ In our previous work,^40^ the structural similarity between the instantaneous and target far fields was shown to remain close to unity over extended operation, demonstrating the temporal stability of the phase-locked array. We therefore anticipate similar fidelity in the elevated-power regime. Crucially, neither route affects the formation of the texture itself, because the polarization topology is encoded in the relative phases between channels rather than in the power those channels carry, the same phase distributions therefore generate the same topology at higher power levels.

The measurements of the optical field topology presented in Figure 5 were performed using a single-camera polarimetry scheme, in which six sequential intensity measurements corresponding to horizontal, vertical, diagonal, antidiagonal, and right- and left-circular polarization components were acquired. This standard technique is robust and enables accurate reconstruction of the Stokes vectors for stationary or quasi-static beams. However, its sequential nature prevents the direct observation of rapid or dynamic changes in the polarization topology.

To demonstrate real-time, programmable tunability of the optical skyrmionic textures in a programmable CBC array, we therefore implemented a four-camera polarimetric system, in which the horizontal, vertical, diagonal, and right-circular polarization components were measured simultaneously using four independent cameras. This configuration forms a parallel imaging polarimeter and enables instantaneous reconstruction of the evolving Stokes-vector field. While this multicamera approach is inherently more sensitive to alignment errors and requires precise pixel-to-pixel registration between the four detection channels, it provides direct access to the temporal evolution of the field topology. Importantly, it allows dynamic topological transitions to be monitored in real time.

Videos S1–S3 illustrate the real-time evolution of the Stokes-vector field, including transitions from Bloch-type to Néel-type textures, from Néel-type to antiskyrmion texture, and the rotation of the antiskyrmion texture, respectively. The closed-loop control system operated at an effective bandwidth of approximately 600 Hz, with about 1 ms devoted to camera image acquisition and 0.5 ms to the NN inference for determining the phase adjustments required to maintain or modify the topology of the optical field. The videos demonstrate clear topological transitions occurring within 2 ms and completed in a single control step, thereby highlighting the degree of programmability and responsiveness of the proposed system. All reported timings were achieved using CPU-based processing (AMD Ryzen Threadripper PRO 7955WX). Further improvements in switching could be achieved through hardware acceleration such as implementing the phase control algorithm on an FPGA and employing a higher-speed camera.

## Conclusion

We have theoretically proposed and experimentally demonstrated that CBC system can be used to generate flexible, on-demand topologically structured optical fields. By treating CBC system as a discrete, electronically addressable optical phased array, we show that the topology of the optical field, including its helicity and skyrmion number, becomes a reconfigurable control parameter rather than a fixed property. This capability is demonstrated through a deterministic and reversible switching between distinct topological states, including Bloch-type, Néel-type, and antiskyrmion textures, using a 7-channel CBC array. Crucially, CBC is intrinsically scalable in both channel number and output power and is inherently modular, providing a pathway toward high-power topological vector fields in which the topology is reconfigured purely electronically, without moving or diffractive optical elements in the beam path. As such, this work establishes topology as a programmable degree of freedom in structured light, bridging topological photonics, high-power laser science, and reconfigurable optical matter.

## Supplementary Material









## Data Availability

The data sets used and/or analyzed during the current study are available from the corresponding author upon reasonable request.

## Notes

Methods for evaluating the skyrmion number; generation of bimeronic textures through CBC; upper limit of the attainable skyrmion number in a CBC array; alternative route to skyrmion formation; dependence of the generated texture on the power ratio of individual emitters within the CBC system (PDF)
Video S1: real-time evolution of the Stokes-vector field; transition from a Bloch-type to a Néel-type texture (GIF)
Video S2: real-time evolution of the Stokes-vector field; transition from a Néel-type texture to an antiskyrmion (GIF)
Video S3: real-time evolution of the Stokes-vector field; rotation of the antiskyrmion texture (GIF)
