# Supplementary material for: Programmable Topological Light via Coherent Beam Combination
Source: ACS Photonics. 2026 Sep 4;13(18):5462–70. doi: 10.1021/acsphotonics.6c01419 (PMC13587772; doi:10.1021/acsphotonics.6c01419)
Supplement: Supplementary file 1 [file ph6c01419_si_004.pdf]

Supporting Information for: **Programmable topological light via coherent beam combination**

Fedor Chernikov,[1,*] William Kerridge-Johns,[1] Konstantin K. Bobkov,[1] Johan Nilsson,[1] Michalis N. Zervas,[1] and Ben Mills[1]

[1]*Optoelectronics Research Centre, University of Southampton, Southampton, SO17 1BJ, UK*
**fc1e21@soton.ac.uk*

1. Skyrmion number evaluation in multi-lobe coherent interference patterns

In tiled-aperture CBC, the total field results from the interference of multiple emitters and therefore contains an extended interference pattern with multiple lobes/fringes. Importantly, with the phase distribution considered in the manuscript the polarisation texture is repeated throughout this interference pattern. For example, the polarisation structure observed in the central lobe is also present in the surrounding side lobes, as illustrated in Figure S1. This results from the coherent interference of individual emitters and means that the polarisation texture extends beyond the central region of the combined beam.

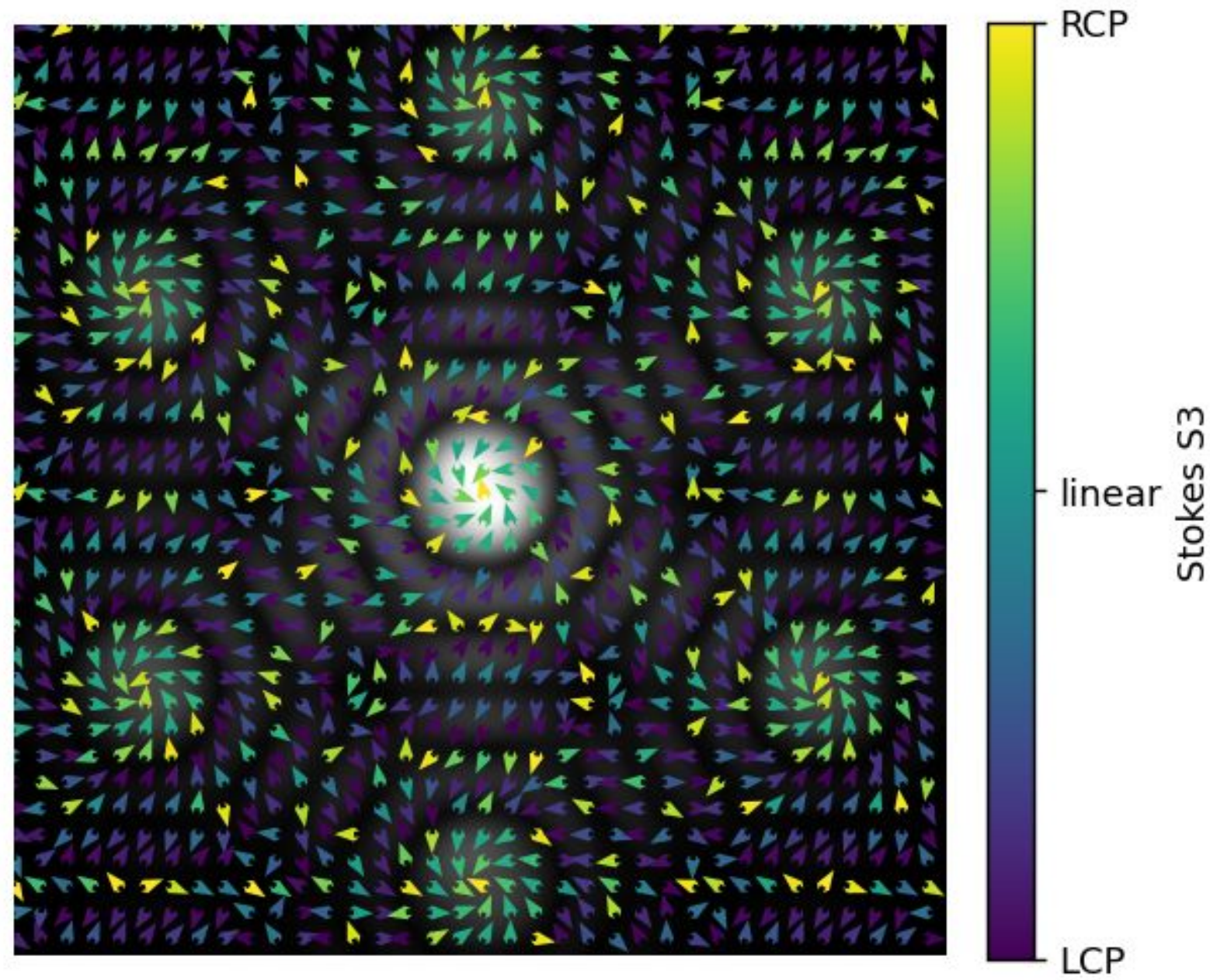

Figure S1. Far-field intensity distribution with the corresponding Stokes vector field overlaid, illustrating the multiple-lobe interference pattern and the repeated polarisation texture.

In numerical and experimental implementations, the integration is necessarily performed over a finite spatial region, and for the CBC field considered here this choice requires particular care. The extended interference pattern contains multiple lobes with a repeated polarisation texture, so an aperture extending beyond the central lobe would accumulate contributions from the neighbouring lobes. The integration region should therefore enclose the central lobe, within which the polarisation completes its excursion across the Poincaré sphere, while excluding the

surrounding structure. We identify this region by evaluating the skyrmion number within a circular aperture centred on the beam axis while systematically varying its radius, and we then verify that a single fixed choice reproduces the expected relationship between the OAM charge (generated by the combined contribution of the outer fibres) and the skyrmion number for all charges investigated.

To determine such a region, we defined a circular aperture centred on the beam axis and evaluated the skyrmion number within this aperture while systematically varying its radius. Figure S2 shows the resulting dependence of the calculated skyrmion number on the aperture radius. As expected, the calculated value depends on the selected integration region. However, there is a range of aperture radii over which the calculated skyrmion number exhibits the desired behaviour. For the simulations presented here, this range is centred around a radius of approximately 48 pixels. With this range, the skyrmion numbers are approximately ($s \approx 1$), ($s \approx 2$), and ($s \approx 3$) for OAM charges ($\ell = 1$), ($\ell = 2$), and ($\ell = 3$), respectively. Similarly, for negative OAM charges, the corresponding values are approximately ($s \approx -1$), ($s \approx -2$), and ($s \approx -3$). This demonstrates that the selected integration region captures the expected dependence of the skyrmion number on the OAM charge.

Importantly, the same fixed integration region is used for all OAM charges, while only phases applied to the outer ring of the fibres are varied to generate the different OAM states. Therefore, the change in the calculated skyrmion number is a consequence of the change in the polarisation structure associated with the OAM charge, rather than a result of changing the integration region. Furthermore, the selected region encompasses primarily the central part of the combined beam, providing a consistent spatial region over which the skyrmion texture can be evaluated for all investigated OAM charges.

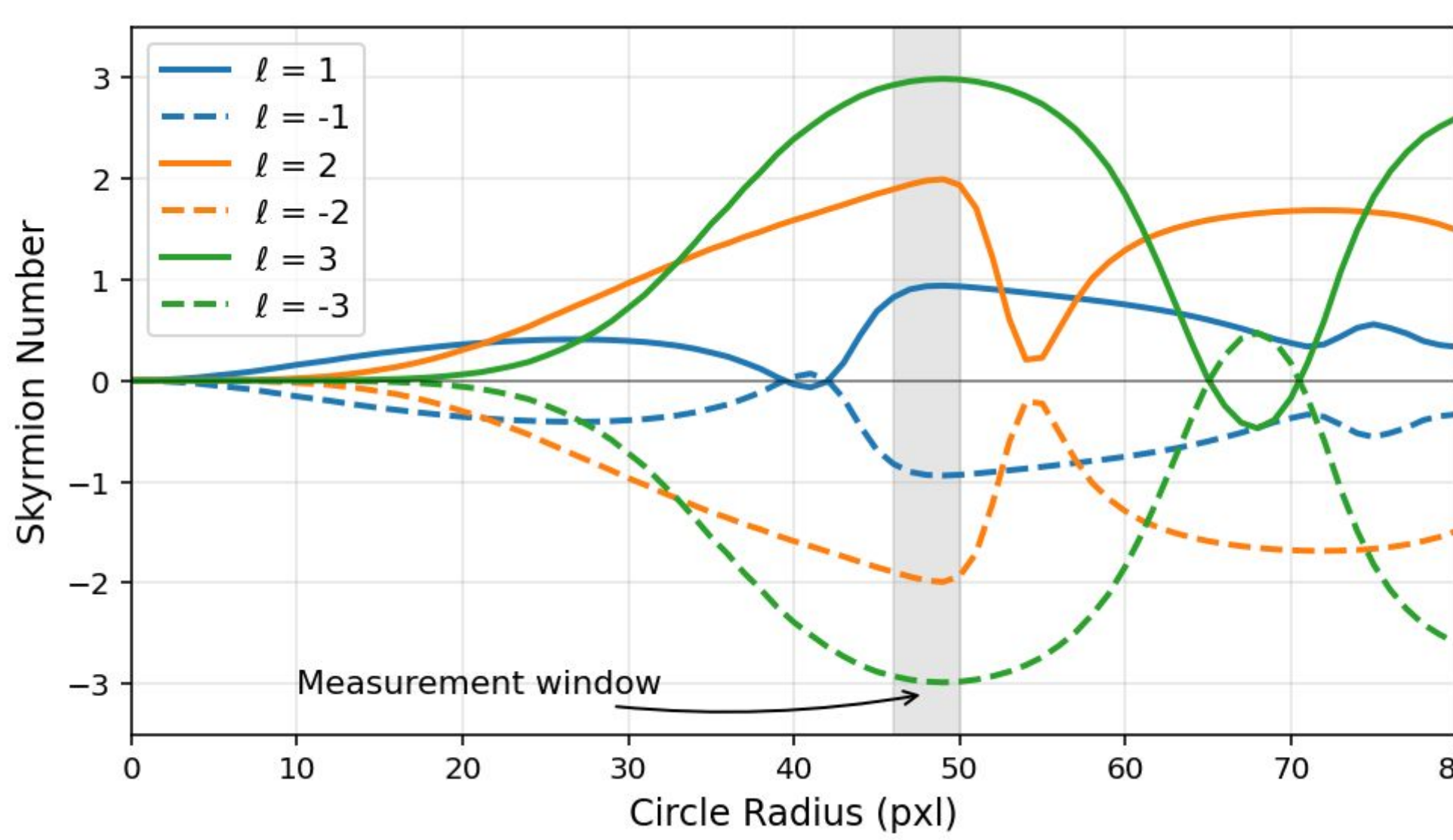

Figure S2 Skyrmion number as a function of the aperture radius in which it is measured. Here ℓ denotes the topological charge that the outer ring of fibres is configured to generate.

Regarding the specific case of ($\ell = \pm 1$), shown in Figure S3, we acknowledge that the polarisation evolution deviates more from being ideally continuous than that for ($\ell = \pm 2$) and ($\ell = \pm 3$), shown in Figure S4(c, d) and (e, f), respectively. For ($\ell = \pm 1$), the polarisation state evolves from right circularly polarised state towards linear polarisation and then through elliptical polarisation before reaching the left circularly polarised region of the polarisation map. Although this evolution is not perfectly continuous, the outer part of the structure contains a range of elliptical and linear polarisation states that contribute to the integrated skyrmion number. This can also be further illustrated by the dependence of the calculated skyrmion number on the radius of the integration aperture shown in Figure S2. As the radius of the circular aperture is increased, different parts of the polarisation texture are progressively included in the calculation. The variation of the skyrmion number with aperture radius therefore reflects the contributions from the different polarisation regions encountered along the radial direction. In particular, as the aperture expands from the central region towards the outer part of the central lobe, the skyrmion number initially approaches approximately (0.5), corresponding to the region where the polarisation evolves from circular towards linear polarisation. As the aperture is further increased and the region of elliptical polarisation is included, the accumulated skyrmion number decreases. It then increases again as the aperture reaches the outer part of the polarisation texture, where the remaining elliptical and circular polarisation, with orientations opposite to those in the central region, contribute to the total skyrmion number. As a result, the final value of the calculated skyrmion number close to unity results from the combined contribution of the entire polarisation texture enclosed by the selected aperture. This demonstrates that, although the polarisation evolution for ($\ell = \pm 1$) is not ideal, the integrated contribution over the selected region nevertheless gives a skyrmion number close to the value of unity.

We attribute the deviation from an ideal continuous polarisation evolution to the imperfect spatial overlap between the generated LG_{00} and LG_{01} modes. We have retained this 37-fibre CBC array because it maintains equal amplitudes across the individual fibre emitters while simultaneously allowing the generation of skyrmion structure with different OAM charges.

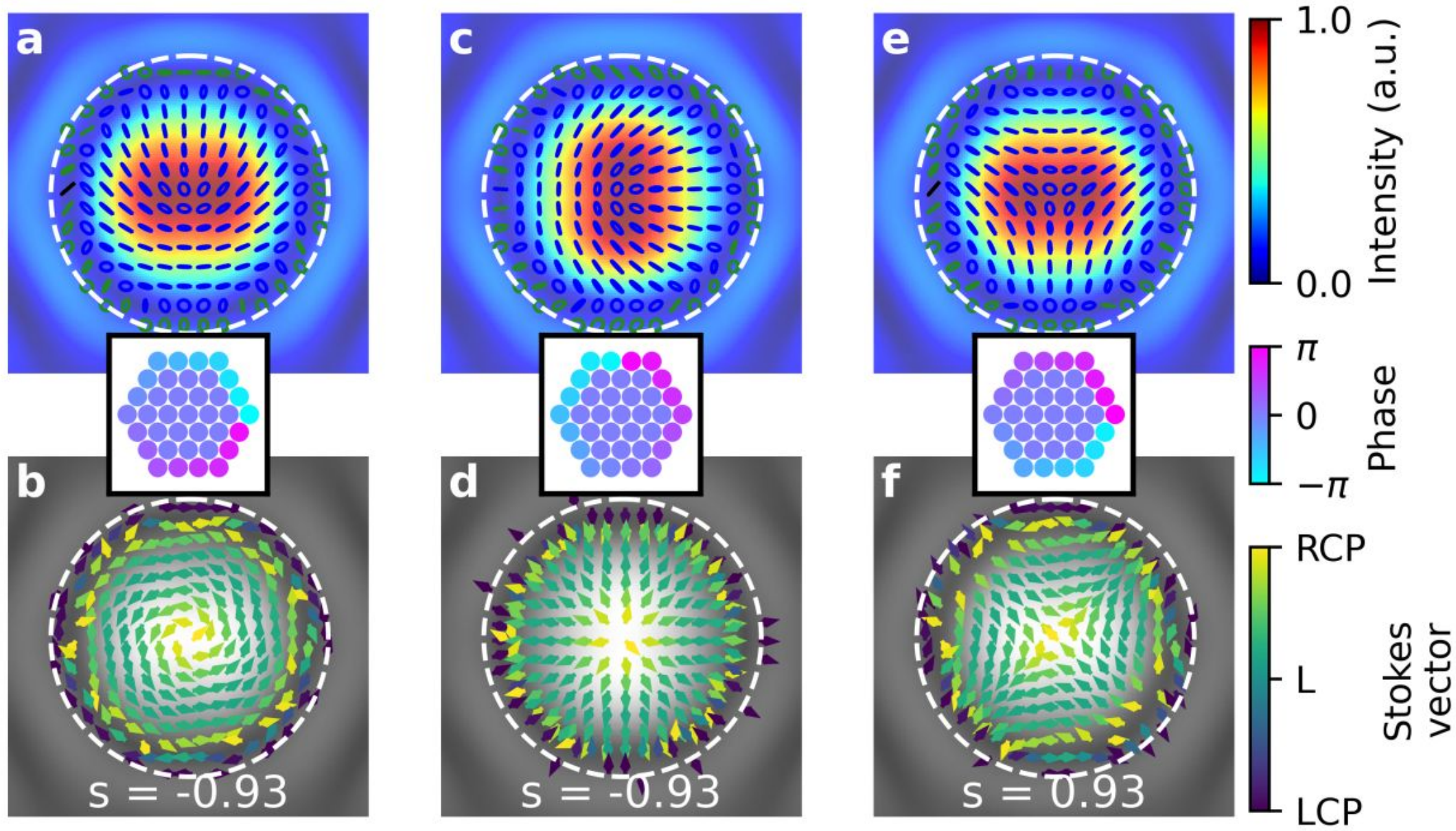

Figure S3 Polarisation textures when outer ring generates beam with OAM charges $|\ell| = 1$. (a, c, e) Polarisation ellipses (where blue and green denote right- and left-handed polarisation) corresponding to Bloch, Néel, and anti-skyrmion textures, respectively. (b, d, f) Corresponding Stokes vector fields. The insets show the corresponding polarisation distributions. The white circles indicate the circular integration region used for calculating the skyrmion number.

This is further illustrated in Figure S4, which shows the polarisation ellipses and corresponding Stokes vector fields for OAM charges $(\ell = 1)$, $(\ell = 2)$, and $(\ell = 3)$. For $(\ell = 1)$, shown in Figure S4(a, b), the polarisation evolution exhibits the non-ideal behaviour discussed above. In contrast, for $(\ell = 2)$ and $(\ell = 3)$, shown in Figure S4(c, d) and Figure S4(e, f), respectively, the polarisation evolves more continuously from right-circular polarisation through linear to left-circular polarisation. Importantly, all three cases are generated using the same 37-fibre array and differ only in the phase distribution applied to the fibres, as indicated in the corresponding insets. Therefore, the non-ideal polarisation texture observed for $(\ell =\pm 1)$ can be considered a trade-off associated with maintaining a common fibre configuration that is compatible with the higher-order OAM states. Nevertheless, as demonstrated in Figure S2, this array still produces skyrmion numbers close to the expected integer values for all investigated OAM charges.

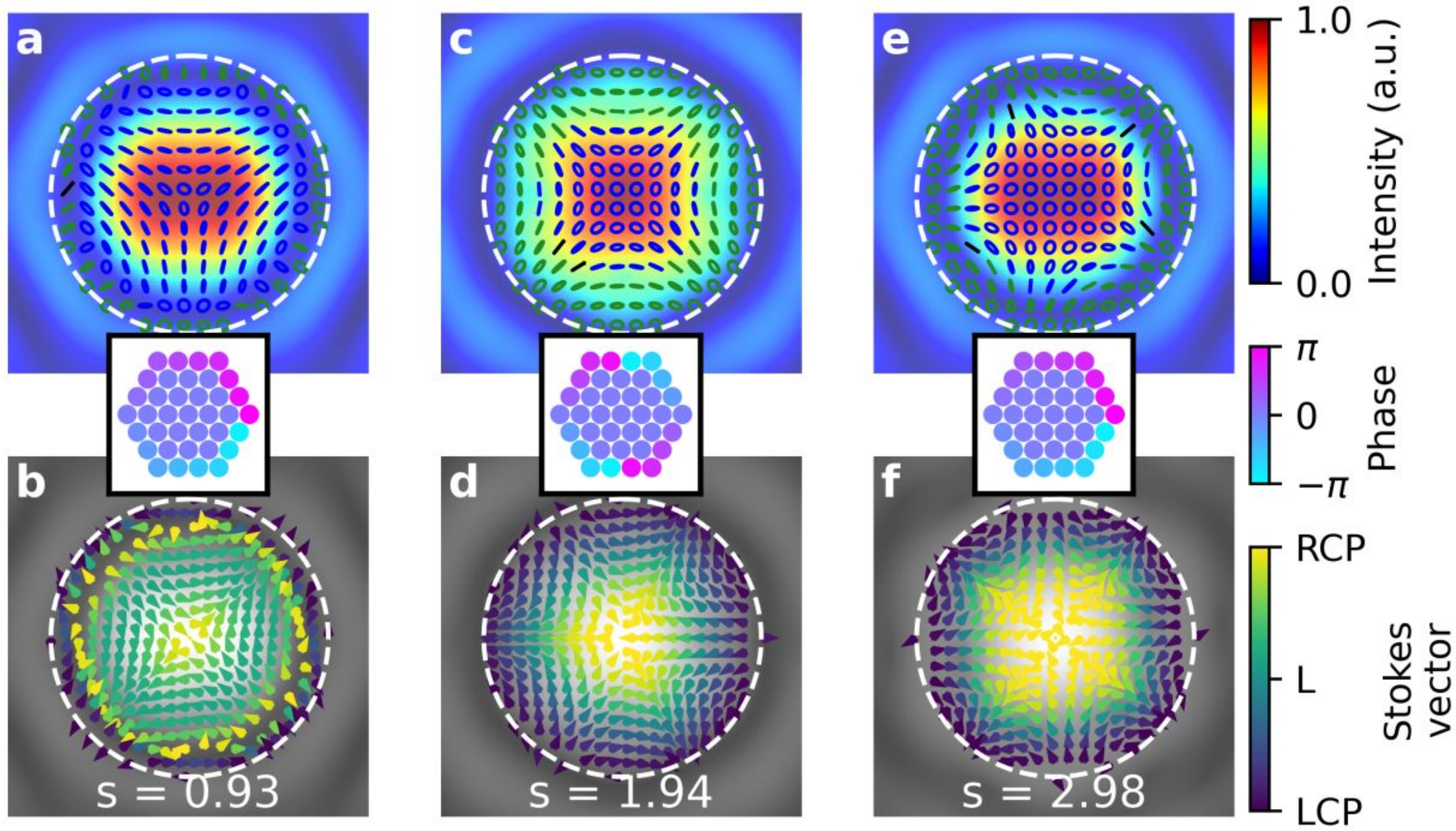

Figure S4 Polarisation textures for anti-skyrmion generated with different OAM charges. (a, b) $\ell = 1$, (c, d) $\ell = 2$, and (e, f) $\ell = 3$. The top row shows the corresponding polarisation ellipses (where blue and green denote right- and left-handed polarisation), while the bottom row shows the corresponding Stokes vector fields. The insets indicate the phase distributions applied to the fibres to generate the respective OAM charges. The white circles indicate the common circular integration region used for calculating the skyrmion number.

2. Bimeronic texture through tiled aperture CBC

Beams with bimeronic textures can be generated with the same technique. A polarisation bimeron is topologically equivalent to skyrmion. The two textures carry the same skyrmion number and are related by a rotation of the Poincaré sphere. In our scheme the skyrmion is generated from two orthogonal circular polarisation states, so the two poles of the texture coincide with the circular-polarisation poles of the Poincaré sphere. Re-assigning the polarisation basis to two orthogonal linear states (e.g. a vertical-linear LG_{00}-like mode and a horizontal-linear $LG_{0\ell}$-like mode) rotates the mapping so that these poles move onto the equator, which is precisely a bimeron.

We confirm this in Figure S5, which shows the resulting bimeron textures, when the outer ring of fibres generates LG_{01}-, LG_{02}-, and LG_{03}-like modes. The polarisation ellipses (top row) and the Stokes-vector field (bottom row) display the characteristic bimeron morphology, while the skyrmion number evaluated over the same analysis region is unchanged from the corresponding skyrmions.

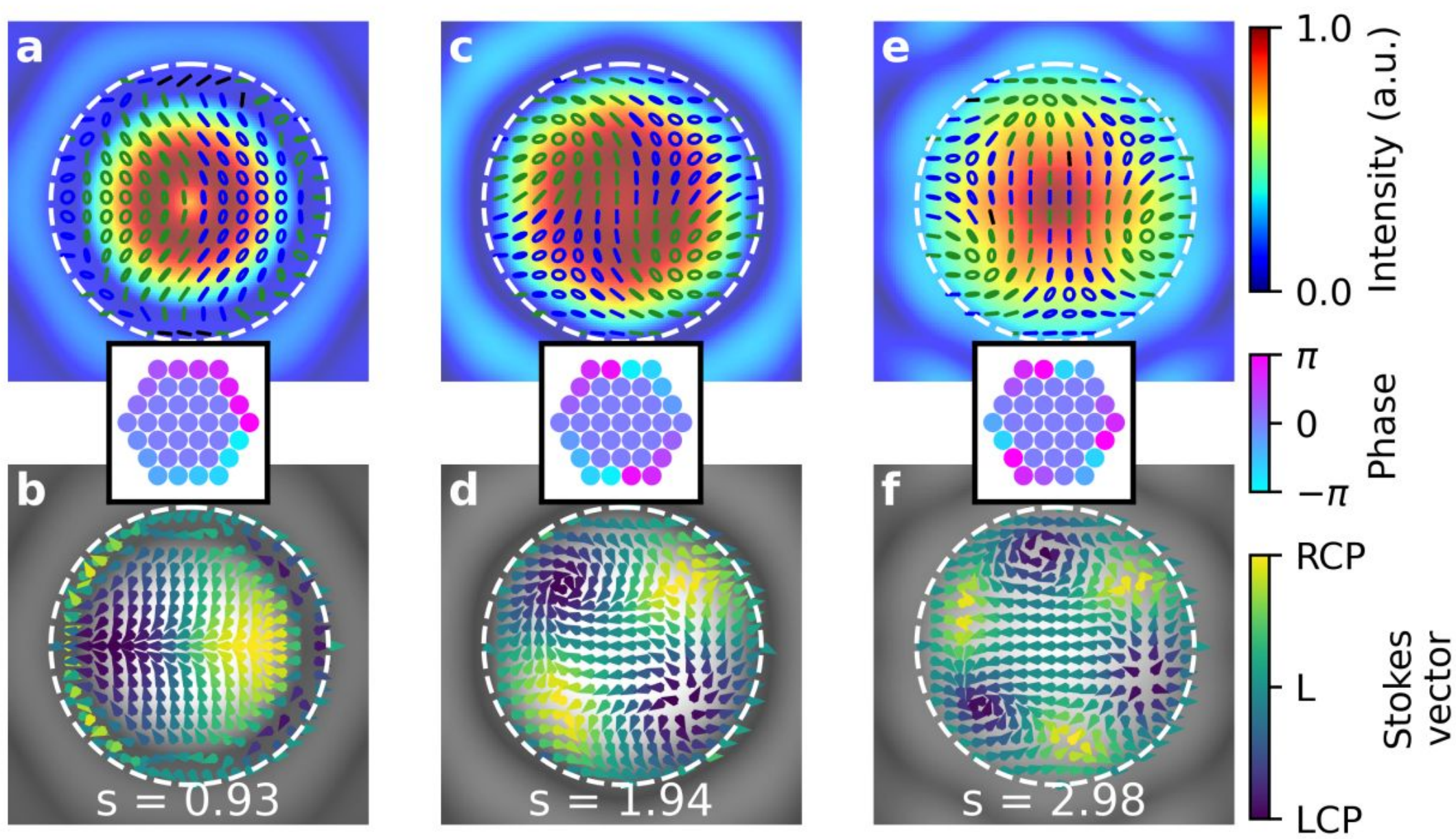

Figure S5 Polarisation textures of optical bimerons generated with different OAM charges. (a, b) $\ell = 1$, (c, d) $\ell = 2$, and (e, f) $\ell = 3$. The top row shows the corresponding polarisation ellipses (where blue and green denote right- and left-handed polarisation), while the bottom row shows the corresponding Stokes vector fields. The insets indicate the phase distributions applied to the fibres to generate the respective OAM charges. The white circles indicate the common circular integration region used for calculating the skyrmion number. The inner 19 fibres are linearly polarised vertically and the outer 18 fibres linearly polarised horizontally.

3. Limit on the attainable topological charge in a discrete emitter array

The limit to the order of the generated optical skyrmion is directly tied to the number of emitters in the CBC array. As with any discretised wavefront-shaping device, this imposes a practical limit.

In the 37-fibre CBC array considered in this work, the inner 19 fibres generate the LG_{00}-like reference mode, while the outer 18 fibres generate the $LG_{0\ell}$-like vortex mode. Generating a charge ℓ vortex requires a total azimuthal phase of $2\pi\ell$ distributed around the outer ring. For $\ell = 3$, for example, this amounts to 6π, i.e. an average of six fibres per 2π, a coarse but faithful discrete approximation of the continuous wavefront. As ℓ increases, the phase step between adjacent fibres grows and the azimuthal sampling coarsens, the ring can represent the vortex faithfully only up to the azimuthal Nyquist limit $\ell = N/2$ (with N the number of the outer fibres), and in practice the texture begins to degrade somewhat before this bound.

We demonstrate this in Figure S6, where the effective charge ℓ imposed on the 18 outer fibres is swept while the amplitude and polarisation state of every fibre are held fixed (as described in the main manuscript) and the skyrmion number is evaluated over the same fixed analysis region established earlier for consistency. The calculated skyrmion number follows the ideal $s = \ell$ line up to $\ell = 5$ and departs from it from $\ell = 6$ onward, demonstrating that a fixed emitter count sets an upper bound on the attainable order.

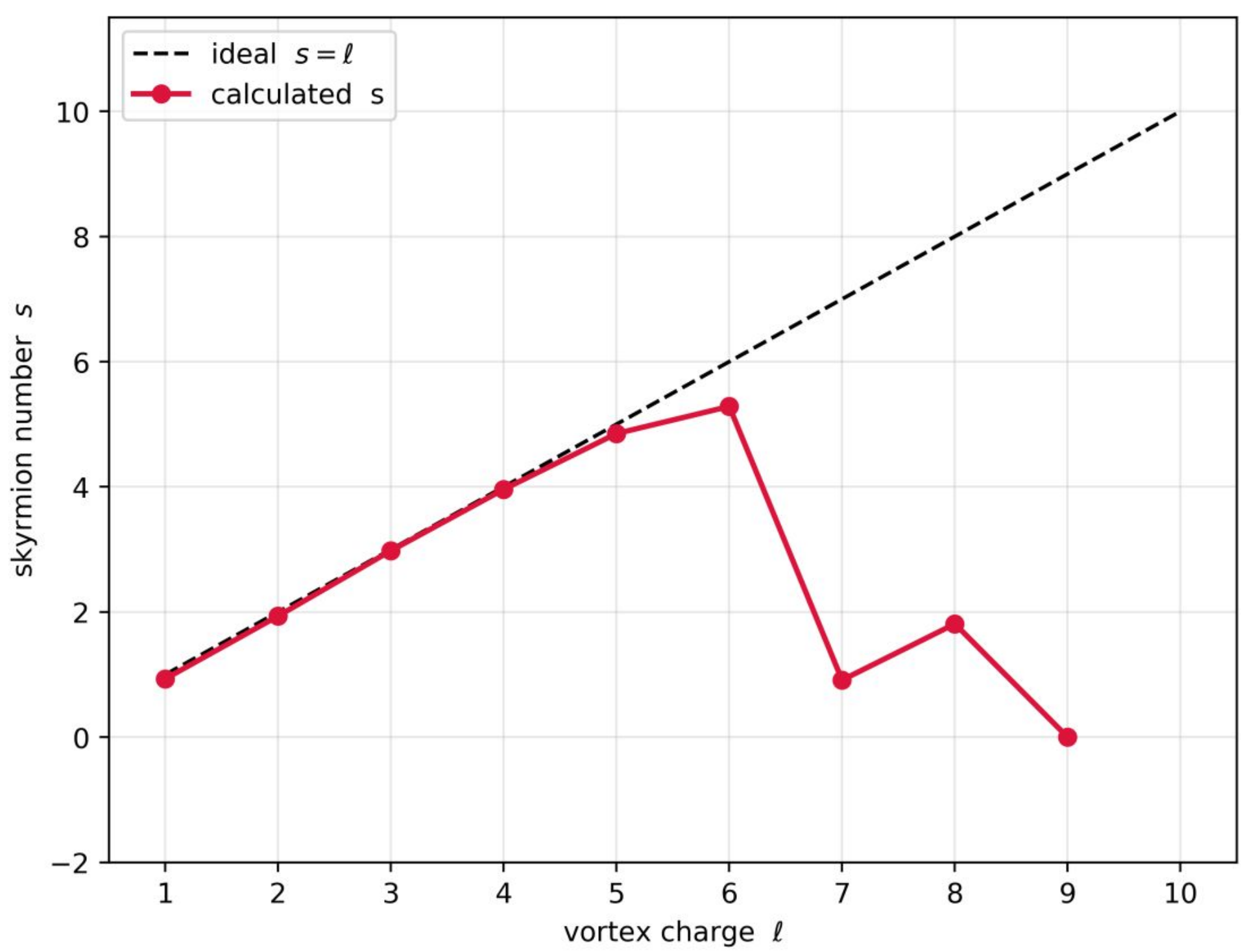

Figure S6 Calculated skyrmion number s versus imposed vortex charge ℓ for the 37-fibre array. The dashed line marks the ideal $s = \ell$.

This bound can be raised by increasing the number of emitters, which improves the azimuthal sampling of the vortex. Importantly, however, a well-formed skyrmion requires not only the correct azimuthal phase but also a spatial match between the LG_{00} and $LG_{0\ell}$ modes. Adding emitters, while improving the azimuthal phase, can disturb the spatial balance, which can in principle be restored by shaping the per-fibre amplitudes, for example through amplitude apodisation, but this is a further degree of complexity that we do not pursue in the present work.

In summary, the achievable skyrmion order is bounded by the emitter count, analogous to the resolution limit of a spatial light modulator, and while no fundamental barrier prevents this limit from being extended with larger, suitably apodised arrays, doing so requires balancing azimuthal sampling against spatial mode matching.

4. Alternative configuration: inner fibres generating the $LG_{0\ell}$-like mode

We have examined the arrangement, in which the inner fibres form the $LG_{0\ell}$-like mode and the outer ring forms the LG_{00}-like mode, and present the results in Figure S7. Figure S7a, b shows the case in which the inner fibres form an $LG_{0\ell}$-like mode. The central fibre is excluded since an emitter located on the phase singularity fills the vortex core and degrades the OAM-carrying mode[1-3]. The polarisation ellipses now evolve continuously from one circular polarisation state at the centre to the orthogonal state at the boundary. Two complications arise, however. First, because the outer-ring fibres are widely separated, the spot they generate is comparatively small, whereas the LG_{01}-like mode is comparatively large; only part of the resulting central spot therefore contributes to the texture. Second, and more restrictively, raising the topological order by phase adjustment alone becomes progressively harder. As shown in Figure S7c,d, the polarisation texture does not complete its evolution within the analysis region: each inner ring contains too few fibres to approximate a continuous helical wavefront once the phase step between adjacent emitters becomes coarse. This constraint is relieved more naturally in our original configuration, where the larger outer ring provides finer azimuthal sampling. Increasing the charge further compounds the difficulty, as shown in Figure S7e,f.

Taken together, these results indicate that the alternative configuration is viable at low order but is not simply a matter of reassigning the mode roles between the two fibre groups; realising it at higher order would require dedicated attention to the radial mode matching and to the azimuthal sampling of each ring. We consider this a promising direction that requires further investigation, but a full analysis falls beyond the scope of the current work.

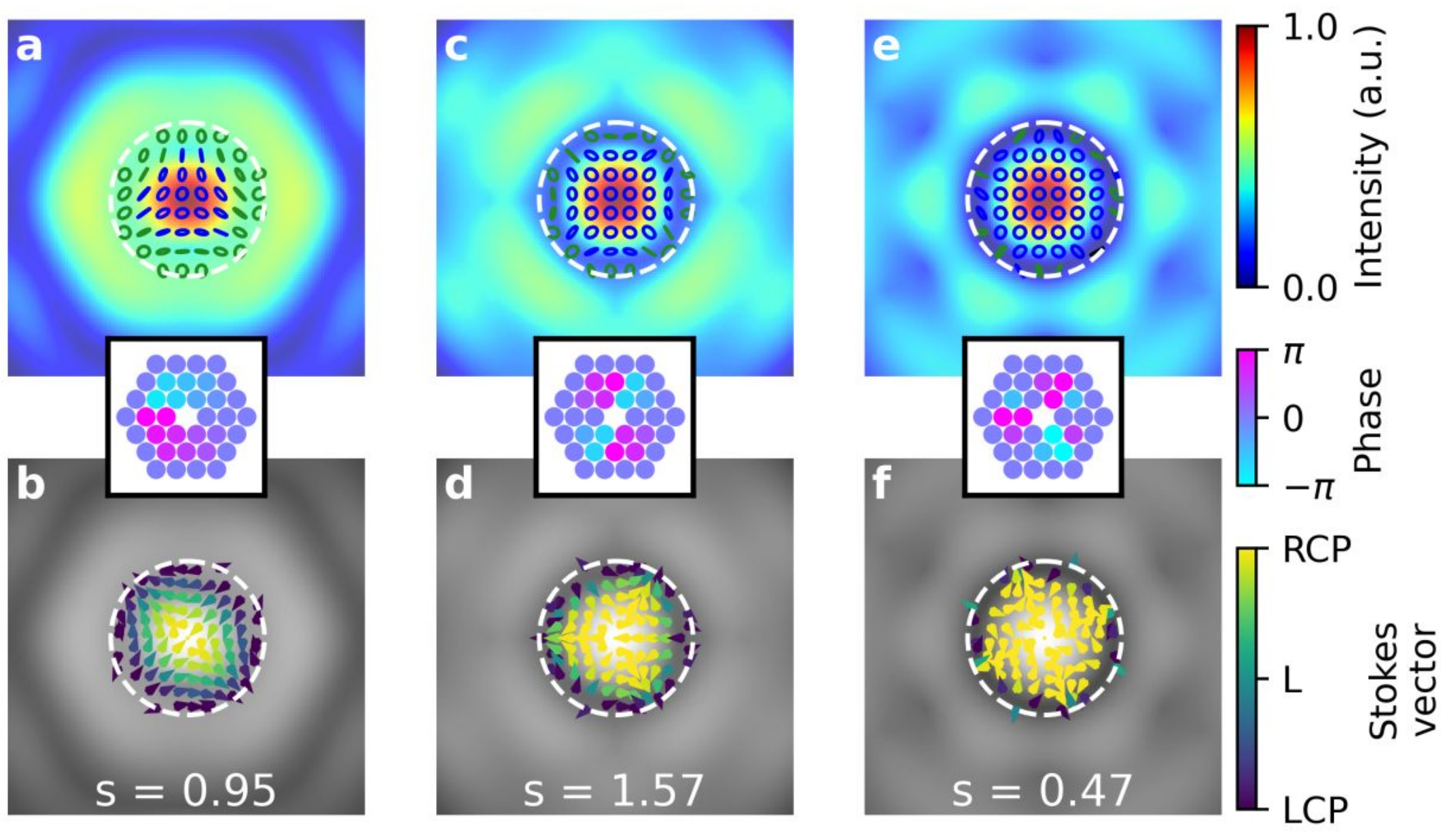

Figure S7 Alternative configuration in which the outer 18 fibres are held in phase to form an LG_{00}-like mode and the inner fibres form an $LG_{0\ell}$ -like mode. Top row: polarisation ellipses (where blue and green

denote right- and left-handed polarisation). Bottom row: Stokes vectors. (a, b) $\ell = 1$; (c, d) $\ell = 2$; (e, f) $\ell = 3$. The white circle indicates the region over which the skyrmion number was calculated.

5. Effect of the inner/outer power ratio in the 7-fibre configuration

In a 7-fibre array the choice of power in the central and outer fibres has a pronounced effect on the combined far field. We therefore examine numerically how the power ratio governs the resulting skyrmion number.

Figure S8 shows the skyrmion number as a function of the outer-fibre power, defined such that every fibre in the outer ring carries the same power, expressed as a fraction of that in the central fibre. The skyrmion number is evaluated over a circular aperture covering essentially the whole of the central lobe of the combined beam, indicated by the dashed circle in each inset. Reversing the sign of the azimuthal phase increment around the ring reverses the sign of the skyrmion number but leaves its magnitude unchanged, so that the phase increment determines the sign of the texture whilst the power ratio determines its magnitude.

When the outer-fibre power is zero the ring makes no contribution, the field is uniformly right-circularly polarised, and the skyrmion number is zero. As the power increases the polarisation texture develops and the skyrmion number grows monotonically, though with a diminishing gradient across the range considered.

At a power ratio of 1/10 the intensity at the centre of the combined beam is largely preserved, retaining about half its peak value, but the polarisation evolves only weakly, remaining right-elliptical as far as the edge of the analysis aperture; the texture therefore covers less than a quarter of the Poincaré sphere.

At the opposite extreme, a power ratio of unity gives a skyrmion number of magnitude approximately 0.74, and the polarisation texture is correspondingly more complete, evolving from right-circular on axis to left-elliptical at the edge of the analysis aperture. The limitation in this case is the intensity distribution: the central intensity falls below a tenth of its peak value, and it is precisely this region in which the central fibre, and hence the right-circular polarisation state, makes its contribution. That the skyrmion number remains well below unity even at the highest power ratio reflects the limited spatial overlap between the LG_{00}-like and LG_{01}-like modes produced by a single ring of six emitters, which cannot be remedied by adjusting the power balance alone.

A ratio of 1/3 provides a compromise between these two regimes. The polarisation reaches the linear state at the edge of the analysis aperture without fully attaining the left-circular state, whilst the central intensity retains about a quarter of its peak value. This ratio was therefore selected for the experimental demonstration presented in the main manuscript.

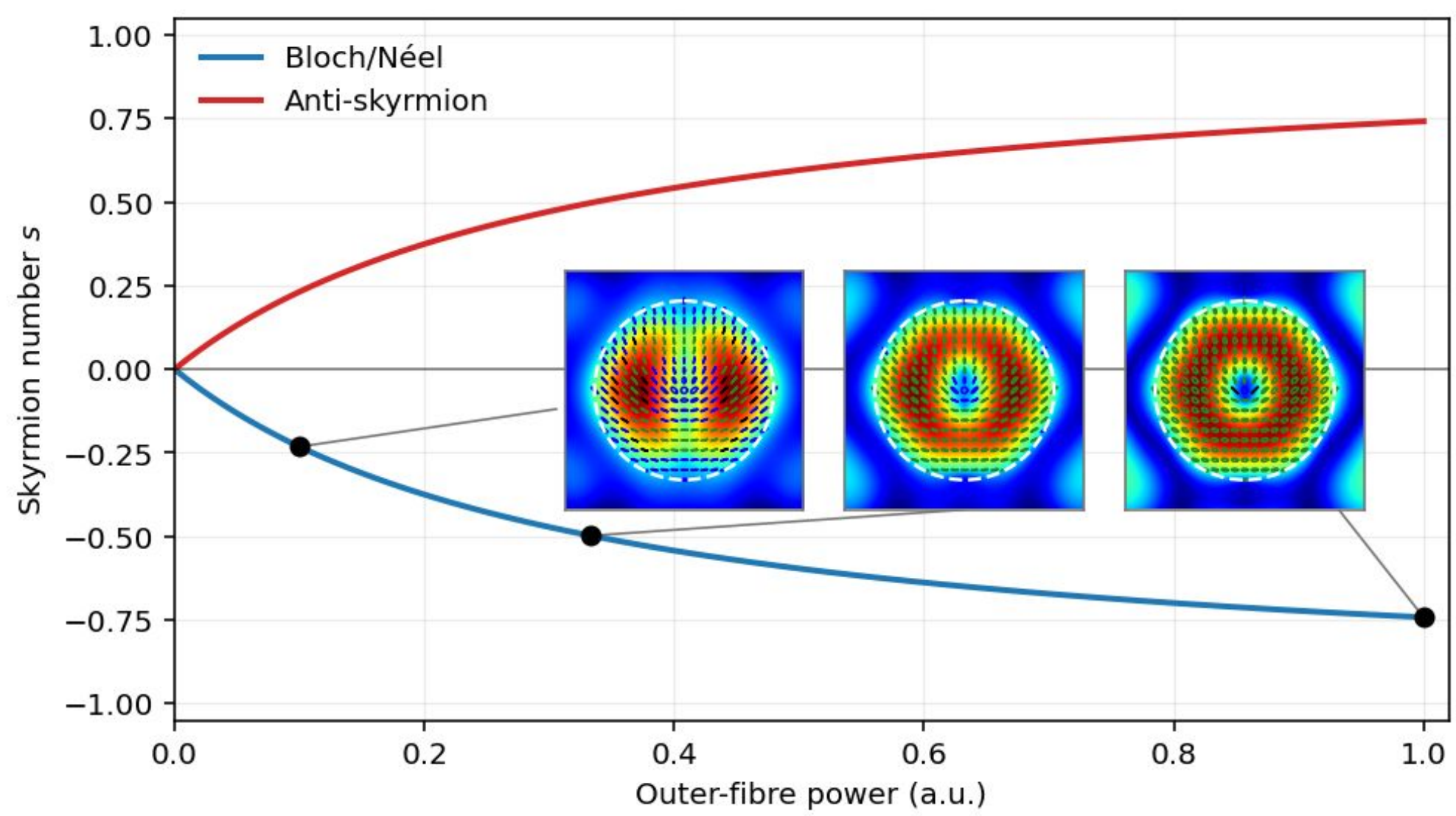

Figure S8 Skyrmion number as a function of power ratio between each outer fibre and the central fibre in the 7-channel CBC array. The two curves correspond to opposite signs of the azimuthal phase increment around the outer ring, giving the skyrmion (Bloch- and Néel-type) and anti-skyrmion configurations. The three insets show the intensity distribution in the central lobe with the polarisation ellipses overlaid (where blue and green denote right- and left-handed polarisation), for outer-fibre power ratios of 1/10, 1/3 and 1, respectively. The dashed white circle marks the circular aperture over which the skyrmion number is evaluated.

Supplementary material reference list

(1) Ma, P.; Zhou, P.; Ma, Y.; Wang, X.; Su, R.; Liu, Z. Generation of azimuthally and radially polarized beams by coherent polarization beam combination. *Opt. Lett.* **2012**, *37* (13), 2658–2660. DOI: 10.1364/OL.37.002658.
(2) Ju, P.; Fan, W.; Gao, W.; Li, Z.; Gao, Q.; Li, G.; Jiang, X.; Zhang, T. Generation of perfect vectorial vortex beams by employing coherent beam combining. *Opt. Express* **2023**, *31* (7), 11885–11898. DOI: 10.1364/OE.485396.
(3) Zhang, W.; Jin, K.; Su, R.; Chang, H.; Ma, Y.; Jiang, Z.; Zhou, P. Controlled generation of arbitrary cylindrical vector beams with an internally sensed coherent beam combining system. *Opt. Express* **2025**, *33* (17), 35374–35384. DOI: 10.1364/OE.569910.
